# Recent advances in titanium dioxide bio-derived carbon photocatalysts for organic pollutant degradation in wastewater

**DOI:** 10.1016/j.isci.2025.112368

**Published:** 2025-04-08

**Authors:** Ethan Dern Huang Kong, Chin Wei Lai, Joon Ching Juan, Yean Ling Pang, Cheng Seong Khe, Irfan Anjum Badruddin, Femiana Gapsari, Khairul Anam

**Affiliations:** 1 Nanotechnology and Catalysis Research Centre (NANOCAT), University of Malaya, Kuala Lumpur 50603, Malaysia; 2 Department of Chemical Engineering, Lee Kong Chian Faculty of Engineering and Science, Universiti Tunku Abdul Rahman, Kajang, Selangor 43000, Malaysia; 3 Department of Fundamental and Applied Sciences, Universiti Teknologi PETRONAS (UTP), Seri Iskandar 32610, Malaysia; 4 Mechanical Engineering Department, College of Engineering, King Khalid University, Abha 61421, Saudi Arabia; 5 Department of Mechanical Engineering, Faculty of Engineering, Brawijaya University, MT Haryono 167, Malang 65145, Indonesia

**Keywords:** Green chemistry, Environmental chemistry, Environmental engineering

## Abstract

Water pollution from organic pollutants such as dyes and pharmaceuticals poses severe threats to ecosystems and human health, demanding effective remediation strategies. Conventional water treatment methods fall short in eliminating these contaminants, prompting interest in photocatalysis, which uses light energy to degrade pollutants into harmless substances such as carbon dioxide and water. This sustainable approach offers efficient pollutant removal with recyclable photocatalysts but faces challenges such as rapid charge recombination and limited electron-hole migration. Research aims to enhance photocatalytic efficiency under UV, visible, and solar light through metal doping and binary oxide systems, particularly titanium dioxide, which improves charge carrier migration and delays recombination. Coupling titanium dioxide with bio-derived carbon shows promise in enhancing electron-hole separation and visible light absorption. This review explores advances in photocatalyst synthesis, degradation mechanisms, adsorption reactions, and economic value of bioderived photocatalysts, emphasizing the potential of photocatalysis for efficient wastewater treatment.

## Graphical abstract

**Figure:**
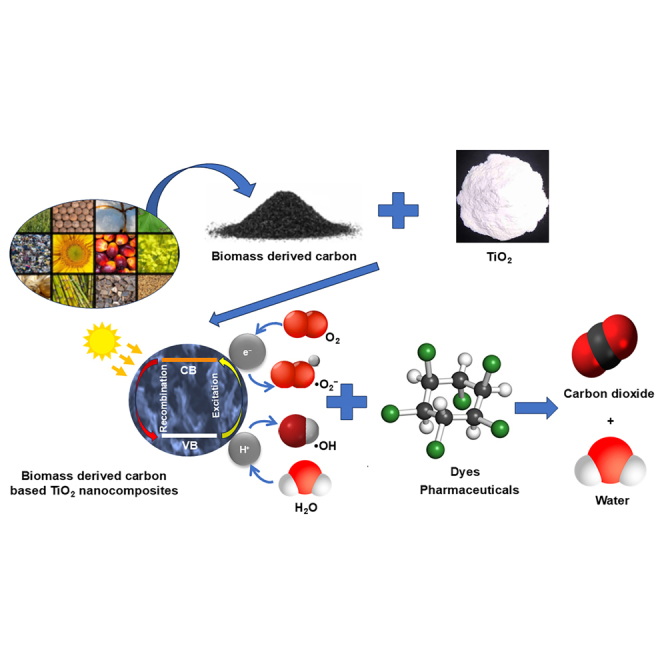

## Introduction

The advancement of human civilization, along with technology, has brought about ecological issues. The increased usage of readily accessible freshwater has led to an increase in wastewater discharge, which has led to an increase in the need for clean water as well as a host of other problems. Statistics revealed an estimate of 80% of industrial wastewater released threatens public health and aquatic biosystems.^1^ Consequently, one major issue faced to date is wastewater remediation due to poor pollution management policies and a vast amount of industrial effluents introduced into water systems. Wastewater generated by anthropogenic activities comprises a significant number of organic pollutants.

**Figure 1:**
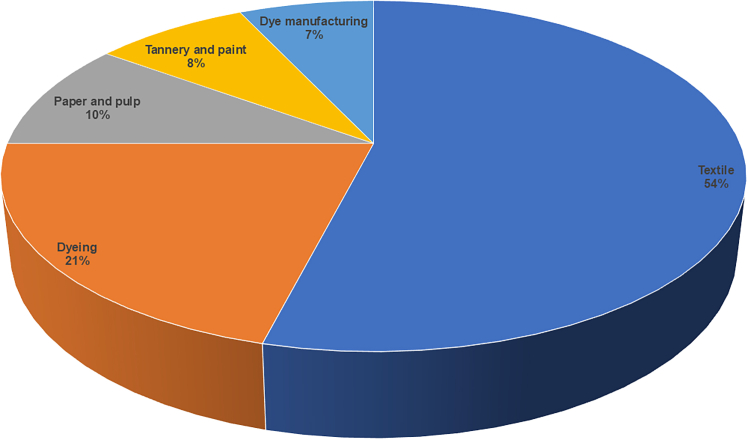
Major industrial contributors for dye pollution

One of the prevalent organic contaminants in water is dyes, and most of these chemical compounds contain intricate organic molecular structures. Azo dyes make up over 70% of all commercial dyes used by the world’s textile industries. Most dyes are hazardous, carcinogenic, and non-biodegradable, which harms both the environment and human health.^2^^,^^3^ About 50% of the synthetic dyes used in the textile industry are considered to not adhere to the cloth and end up being released into the environment along with others sources as in Figure 1.^4^ Synthetic dyes in water are detrimental to nature because they prevent light from penetrating, prevent aquatic photosynthesis, and disrupt the entire biological system as a result. Furthermore, it has been noted that exposure to this dye pollution by humans through food chains is exceedingly hazardous and frequently lethal.^5^ Dye residues in the soil have a negative impact on plant and animal health as well as soil fertility.

Apart from that, pharmaceuticals are a class of organic products formed for use as medicines to treat diseases, which have improved lifespan and human health. By triggering genetic exchange, antibiotics released into the environment, for instance, may have long-lasting and irreversible impacts on microbes. This would exacerbate the development of pathogen resistance to a variety of antibiotics.^6^

**Figure 2:**
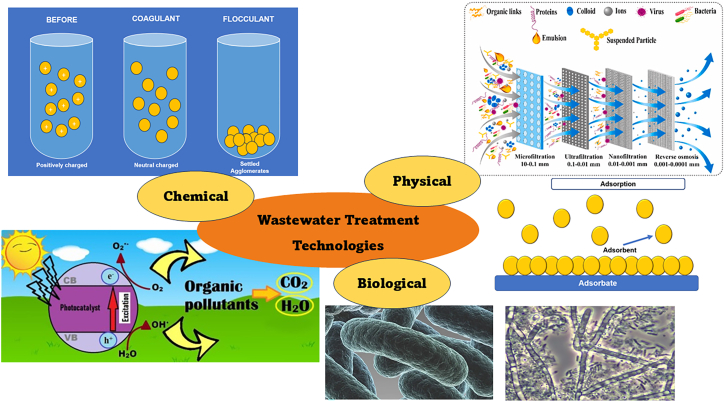
Conventional methods for wastewater treatment

Different approaches and techniques were used for the removal of organic pollutants from wastewater as in Figure 2. Physical methods (sedimentation, filtration, and adsorption) involve mass transfer of pollutants from different mediums. Biological methods remove pollutants with microorganisms that are capable of breaking down pollutants through biological oxidation or by biosynthesizing microbial cells using organic pollutants in wastewater with dense biomass and are eventually removed by sedimentation.^7^ Chemical methods employ chemicals that can agglomerate pollutants to form larger clumps (coagulation and flocculation) for later removal or advanced oxidation processes that induce radicals to break down organic pollutants into simpler compounds. As a standalone, different methods have advantages and drawbacks that limit their practical use. For example, physical methods are simple and straightforward, and yet they only increase the concentration of pollutants instead of removing them. Biological methods can be cheap to operate, but the process is slow, has low biodegradability, and requires an optimal environment.^8^^,^^9^ Chemical methods can rapidly oxidize and completely degrade pollutants but are only done on a laboratory scale.^10^ Regardless of the advantages of these methods, as a standalone, they have restricted efficiency due to their drawbacks. Recently, hybrid treatment methods involving a combination of these methods were promising. Adsorption combined with photocatalysis had stood out in water remediation not only from its high efficiency but also its economical and eco-friendly.^11^^,^^12^^,^^13^

**Figure 3:**
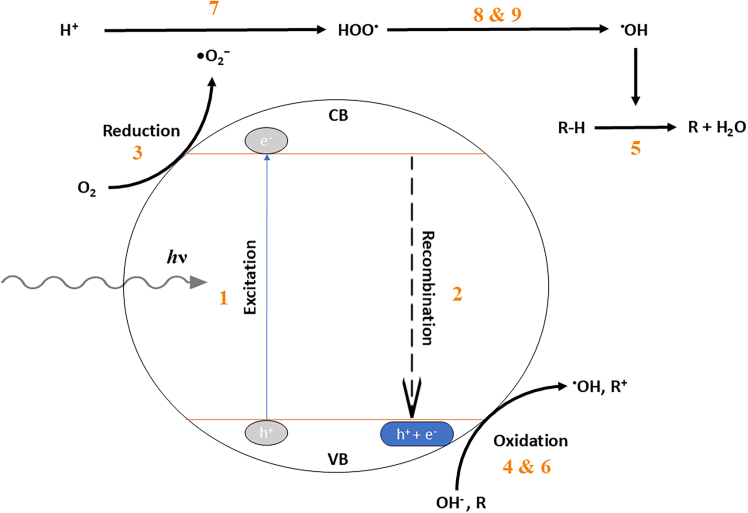
Schematic diagram of photocatalytic degradation

Photocatalysis commonly involves a catalyst irradiated by a light source and speeds up the rate of chemical reactions. Heterogeneous photocatalysis is an advanced oxidation process that is suitable for wastewater remediation for its capability to remove organic pollutants while utilizing natural and renewable solar energy. An efficient photocatalyst would need the following processes to occur (i) light irradiation (irradiation energy greater or equivalent to the bandgap of semiconductor which excites electron from valence band to conduction band), (ii) exciton separation, (iii) movement of charge carriers without recombination in the bulk or on the surface of the photocatalyst and lastly, (iv) redox reactions between radicals induced by charge carriers and target pollutants on the surface of photocatalyst.^14^ The outcome of the process brings down the toxicity of wastewater to an acceptable concentration prior to its release into water streams. Organic pollutants are either oxidized or reduced into harmless products such as carbon dioxide (CO_2_) and water (H_2_O), which consequently decreases secondary pollutions as shown from Equations 1, 2, 3, 4, 5, 6, 7, 8, and 9 and Figure 3.

(1) Photocatalyst + ℎν → Photocatalyst (e^−^+ h^+^)

(2) e^−^+ h^+^ → Heat

(3) e^−^ + O_2_ → ·O_2_^−^

(4) h^+^ + OH^−^→ ·OH

(5) ·OH + R−H → R + H_2_O

(6) h^+^ + R → R^+^ → Intermediates

(7) e^−^ + O_2_ → ·O_2_^−^ + H^+^ → HOO· + ·O_2_^−^ → HOO·+ O^−^

(8) HOO· →H_2_O_2_ + O_2_

(9) H_2_O_2_ + ·O_2_^−^ → ·OH + OH^−^ + O_2_

**Figure 4:**
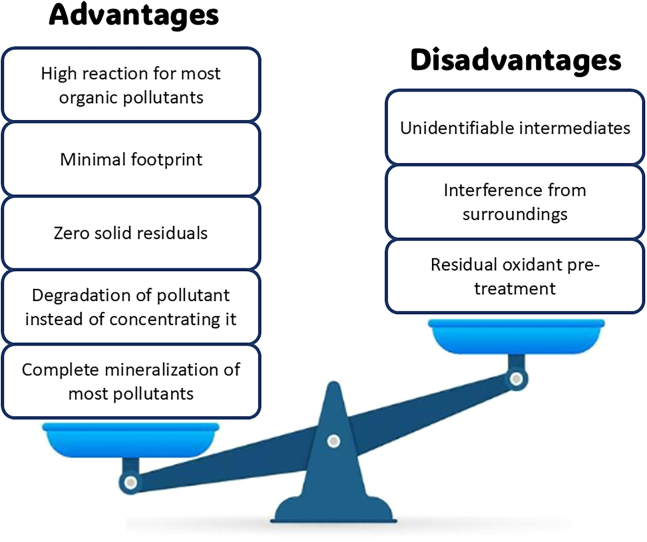
Merits and shortcomings of photocatalysis for organic pollutants

Heterogeneous photocatalytic processes are generally cheap, non-toxic, and environmentally sustainable to degrade recalcitrant contaminants under mild conditions.^15^ Photocatalysts can also be recycled for future use as they are not exhausted throughout the process and could be versatile in many wastewaters with organic pollutants.^16^ Figure 4 summarized the advantages and disadvantages of photocatalysis.^17^ The study for semiconductor photocatalysts that can effectively utilize solar energy for effective energy consumption remains a huge problem even though photocatalysis has attracted enormous attention in environmental remediation, photosynthesis, and water splitting. Hence, determining the best and most promising photocatalyst for the removal of dyes and organic pollutants has thus been the subject of countless studies and modifications.

To date, nanoparticles such as zinc oxide (ZnO), tungsten oxide (WO_3_), iron oxide (Fe_2_O_3_), and titanium dioxide (TiO_2_) were utilized as semiconductor photocatalysts.^18^ ZnO with a bandgap of 3.2eV is also categorized as an n-type semiconductor. ZnO photocatalysts are highly photosensitive, cheap, and environmentally friendly. However, the application of ZnO is restricted as it shares similar drawbacks with TiO_2_. WO_3_ has a narrower bandgap of 2.8eV as compared to TiO_2_ and ZnO, which makes it more suitable for visible light adsorption. Despite having a lower bandgap than the mentioned photocatalysts, WO_3_ still suffered from low photocatalytic activity due to the rapid recombination of photoinduced excitons and the conduction band of WO_3_ being more positive than the reduction potential for O_2_/O_2_^−^, thus restricting the reduction of oxygen molecules during the degradation process.^19^^,^^20^ Fe_2_O_3_ photocatalysts with a bandgap from 2.0 to 2.2eV make it suitable for visible light adsorption. Its antiferromagnetic property allows high recovery rates when an external magnetic field is applied. The abundance of hematite in nature makes it an economical photocatalyst. Even so, the position of the valence band and conduction band of Fe_2_O_3_ photocatalysts are at the positive potential with respect to hydrogen production potential, hindering the production of hydrogen.^21^ Further advantages and disadvantages of these photocatalysts are summarized in Table 1.

**Table 1:** Conventional semiconductor photocatalysts for wastewater remediation

Photocatalysts	Advantages	Disadvantages	Reference
ZnO	• Photosensitive • Low toxicity • Low cost	• High recombination rate of electron-hole pairs • Limited response toward visible light and photocatalytic activity	D. Zhu and Q. Zhou^22^
WO_3_	• Responsive toward visible light • Stable in acid medium	• High recombination rate of electron-hole pairs • Limited photocatalytic activity	D. Zhu and Q. Zhou^22^
CeO_2_	• UV and visible light active • Excellent redox properties	• Limited photocatalytic efficiency • Expensive	Tran et al., and Han et al.^23^^,^^24^
CuO	• Narrow bandgap for visible light absorption • High conductivity	• Susceptible to photocorrosion • Moderate activity compared to TiO_2_	Sibhatu et al., and Chen et al.^25^^,^^26^
NiO	• Visible light active • High chemical stability	• Lower photocatalytic activity • Complex synthesis methods	Makhado et al., and Lahiri et al.^27^^,^^28^
SnO_2_	• High transparency and wide bandgap • Good stability	• Inefficient under visible light • High charge recombination	Sun et al., and Ren et al.^29^^,^^30^
Cr_2_O_3_	• High stability in harsh conditions	• Low photocatalytic efficiency • Toxicity concerns	Sompalli et al.^31^
ZrO_2_	• High thermal stability • Non-toxic and durable	• High bandgap, limiting visible light absorption • Moderate photocatalytic performance	Aldeen et al.^32^
Fe_2_O_3_	• Easy recovery, photo-responsive toward solar spectrum	• High recombination rate	Fawzi Suleiman Khasawneh and Palaniandy and Hitam and Jalil^21^^,^^33^
TiO_2_	• Durable • Cheap • Low toxicity • Chemically and photochemically stable	• High recombination rate of electron-hole pairs • Limited response toward visible light and photocatalytic activity	Zhu and Zhou and Mohadesi et al.^22^^,^^34^

TiO_2_ had gained notable attention among these accredited to its chemical stability, nontoxicity, and low cost. Regardless, the applications of TiO_2_ are restricted by its large bandgap, making it irresponsive toward the solar spectrum excluding UV light. Efforts to enhance the photosensitivity of TiO_2_ were done by doping with transition metals (Zn, Ag, Fe, Ni and Cu)^35^^,^^36^ together with non-metals (B, C, N, O and F),^37^ still, expensive manufacturing cost, non-recyclable and the risk of secondary pollution are problems need to be dealt with prior to upscaling. Consequently, attention has been shifted to emphasize the integration of supporting materials, which increases the recoverability rate and photosensitivity toward the rest of the solar spectrum for stable photocatalysts. The combination of TiO_2_ with carbon nanomaterials has been extensively studied due to the synergic effect of these two exhibits as a composite.^38^^,^^39^ The following are the causes: First, carbon has a large specific surface area, excellent pore structure, and strong adsorption properties that can increase the synergistic effect with TiO_2_ to increase photocatalytic activity. These properties can also provide sufficient reaction sites for TiO_2_ and reduce the agglomeration of TiO_2_. The surface charge of the oxide in the composite will grow due to carbon’s high electron uptake and conduction capabilities, which may lessen the likelihood of electron-hole recombination during photocatalysis.

The agriculture sector generates 5 billion metric tonnes (Mt) of biomass waste each year, and that number keeps going up. These unused resources can be found as leftover crop stalks, leaves, roots, and seeds. 77% of the world’s biomass is produced in the Asia region.^40^ The use of waste materials introduces the idea of a "circular economy," which makes it possible to employ waste and recycling for more environmentally friendly industrial goals such as wastewater treatment. Many recent studies have focused on the application of biowaste as well as nature-derived precursors for valuable carbon structures, which make use of available sources that are environmentally friendly and cost effective.^41^^,^^42^^,^^43^^,^^44^ These materials are also promising due to the fact that they can be recycled and sustainable in comparison to conventional carbon-rich precursors such as polymers, organic complexes, and carbohydrates.^45^ Due to the dual environmental remediation of waste management and water purification with no harmful or carbon footprints, the application of carbon materials obtained from waste biomass sources is encouraging for the treatment of wastewater.^44^ At the same time, the demand for primary resources might be significantly decreased by recirculating materials and then employing them more effectively in products, hence reducing environmental impacts. Hence, the substitution of expensive precursors with natural and industrial carbonaceous wastes as carbon precursors would be beneficial to the economy and environment.^46^

According to the literature, studies on the removal of organic pollutants via biomass-derived carbon nanomaterials coupled with TiO_2_ photocatalyst are still at the infancy stage. To evaluate potential alternative sources for carbon nanomaterials, we provide an overview of several fundamental aspects. These aspects are scientifically significant and require further clarification to drive meaningful progress and applications. This review will be divided into several sections: 1. Introduction of industrial dyes; 2. Introduction of pharmaceuticals; 3. Advanced oxidation processes and photocatalysis; 4. Synthesis of bioderived carbon coupled with TiO_2_; 5. Summaries of the photodegradation of organic pollutants by biowaste derived carbon materials coupled with TiO_2_; 6. Economic perspective of biocarbon in photocatalysis; 7. Conclusion and future work.

## Organic pollutants

### Industrial dyes

**Figure 5:**
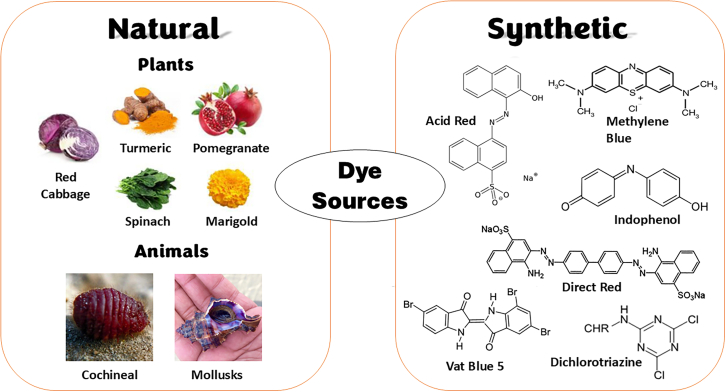
Dye sources

The origins of coloring dye could be naturally derived or synthetic organic compounds from industry as shown in Figure 5. Dyes are distinguished according to chromophores and auxochromes within their complex organic molecules. Chromophores are responsible for each distinct dye color and are comprised of heteroatoms such as nitrogen (N), oxygen (O), and sulfur (S) with non-bonding electrons.^47^ In contrast, auxochromes donate electrons and intensify dye color by enhancing solubility and binding to the fiber.

Acid dyes are anionic, water-soluble dyes used for protein fibers such as silk, wool, and nylon, requiring a low pH for vibrant, durable coloration.^48^ Basic dyes, in contrast, are cationic and best suited for synthetic fibers such as acrylic and polyester, applied in high-pH environments to produce intense colors. Reactive dyes form covalent bonds with cellulosic fibers such as cotton and rayon, offering excellent wash and light fastness.^49^ Direct dyes, also for cellulosic fibers, can be applied directly without a mordant,^50^ forming hydrogen bonds for good wash fastness, though with lower light resistance. All these dyes are widely used in the textile, paper, leather, and ink industries.

### Toxicity of dyes

Organic dyes pose risks to the socioeconomic and environmental aspects of the community as they are carcinogenic and mutagenic when being dumped each year in large quantities.^51^ The presence of dye on the surface of water negatively affects the aesthetic quality and organisms below water by preventing sunlight from penetrating the water's surface.^52^ Acidic and azo dyes have detrimental effects on the gastrointestinal tract, eyes, respiratory system, skin and may cause cancer and mutagenicity in humans. They may also cause enzymatic abnormalities. Azo dyes have an amine group, which is mostly to blame for their toxicity. Basic dye also harms human health by causing laryngitis, mutations, skin cancer, an increase in the incidence of shock, jaundice, neurotoxicity, cyanosis, and tissue necrosis, in addition to skin, allergy, reproductive, and development complications.^53^ When processing textiles, the textile industries use chemicals that include metal ions, particularly when using metal-based mordant dyes. These metal ions, which are typically carcinogenic and have an impact on aquatic life, are released into the environment through the effluent of textile industries.^54^ Water soluble reactive dyes are also quite harmful to the ecosystem. Synthetic food dyes that are consumed directly have substantial side effects that impair the proper operation of several bodily organs. Most dyes are mutagenic, carcinogenic, and can harm different human organs. These dyes also are resistant without treatment and can remain in water bodies for an indefinite amount of time, making them dangerous toward the marine ecosystem. Hence, the removal of organic dyes from wastewater is essential for wastewater treatment today.

### Pharmaceuticals

**Figure 6:**
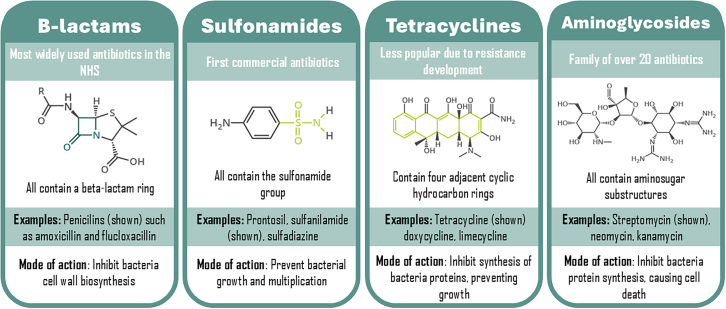
Antibiotic classes

Pharmaceuticals are a broad category of biological chemicals used to treat illnesses and infections. The number of medications found in water bodies, including estrogen, birth control hormones, and painkillers, is extremely alarming.^55^ Pharmacologically active pollutants produced by pharmaceuticals are resistant to degradation and persistent in aqueous media. Pharmaceuticals are target-specific chemicals that are produced to absorb and circulate within the human body. Pharmaceuticals have variable structural characteristics as shown in Figure 6.^56^ The use of pharmaceuticals is influenced by a few variables, including the socioeconomic status of a nation, location and region, access to healthcare, seasonal change, and so forth..^57^ There will be more pharmaceuticals in waste streams because of increased usage of any medicine during a pandemic. Over the past few decades, there has been a significant growth in the manufacturing and consumption of pharmaceuticals, which has resulted in a sharp rise in the content of pharmaceuticals in wastewater. Pharmaceuticals can interact with and be absorbed by living things, which makes them a possible threat to the ecosystem.^58^ The pharmaceuticals penetrate the environment and disrupt the ecosystem as hospital effluents (from hospitals), industrial discharges (from pharmaceutical businesses), agricultural runoffs (pesticides and fertilizers), and human and animal excreta (from homes and sewers).^57^^,^^59^ Hazardous chemicals, solvents, active pharmaceutical ingredients, metabolites, disinfectants, and heavy metals are examples of hospital effluents that can persist in the environment for a very long time, pose major hazards to the environment, and have high liquid phase mobility.^60^^,^^61^ Before releasing these effluents into water bodies, it is crucial to treat them effectively. Pharmaceuticals can be categorized or grouped according to their therapeutic uses. To treat and remove pharmaceuticals from wastewater, a variety of physiochemical and biological treatment techniques are used. The optimal removal strategy for these pharmaceuticals will be developed with the aid of the identification of these classes or groups of pharmaceuticals found in the wastewater.

Based on their mode of action (binding and acting against their biological target), mechanism of action (binding to and acting against their biological target), chemical structures, and the treatment of disorders, pharmaceuticals are divided into various types or groups. Pharmaceuticals are categorized as therapeutic classes or groups when their curative or remedial usage (the pathology they intend to treat) is taken into consideration. These chemicals can be classified as antibiotics, antivirals, antidepressants, contraceptives, hormones, and analgesics.^62^^,^^63^

### Toxicity of pharmaceuticals

There is sufficient evidence to demonstrate that antibiotic residues can negatively affect the structure and functioning of microbial communities.^64^^,^^65^^,^^66^ Antibiotics typically have negative effects on the ability of microalgal cells to photosynthesize, as well as cell proliferation and growth. They can also hinder the formation of chloroplasts, the generation of chlorophyll, and the synthesis of proteins.^67^ Macrolides (erythromycin and roxithromycin) disrupt thylakoid membrane protein synthesis and chloroplast gene translation process,^68^^,^^69^ at the same time, roxithromycin and clarithromycin decrease chlorophyll at the cellular level.^70^^,^^71^ A toxicity study of tetracycline was conducted on *Stentor coeruleus* and *Stylonychia lemnae ciliates and found out that the tetracycline inhibited antioxidant enzymes activity, retarded growth and damaged the ultra-structures of ciliate cells*.^72^ Tetracycline even with its two metabolites, anhydrotetracycline and epitetracycline, were found to have toxicological effects toward *Chlorella vulgaris* by growth retardation, cell permeability variation and oxidative stress.^73^

According to published research, a variety of aquatic organisms and other organisms that ingest these organisms may experience hazardous effects because of pharmaceuticals bioaccumulating in the tissues of marine organisms.^74^^,^^75^ It was discovered that tetracyclines, erythromycin, and norfloxacin all increased mortality as well as the proliferation and digestive activity of lipase, pepsin, and trypsin while weakening the sea cucumbers' immune defenses.^76^ The acute and chronic exposure of erythromycin to *Oncorhynchus mykiss* disturbed its antioxidant defense system, and the resulting increase of reactive oxygen species caused lipid peroxidation (oxidative stress), making erythromycin genotoxic.^77^

Tetracyclines can cause chromosomal abnormalities and plant growth inhibition, which lowers the amount of photosynthetic chlorophyll and carotenoid pigments in plants. Some aquatic plants intended for human consumption that have previously received pig dung fertilizer are bioconcentrated with oxytetracycline.^78^ Additionally, it has been demonstrated that the antibiotics chlortetracycline and tetracycline can alter the enzymatic activities of the earthworm *Eisenia fetida* (superoxide dismutase and catalase), as well as cause DNA damage.

Several studies have examined the toxicity of NSAIDs on aquatic vertebrates. For example, zebrafish embryos were exposed to diclofenac and ibuprofen drugs between concentrations of 0.04 and 25.0 mg/L which resulted in the impairment of cardiac physiology.^79^ Ibuprofen had disrupted cardiac functions by increased blood flow and decreased blood density, while diclofenac prevented the contraction of muscle and reduced hatch rate of zebrafish embryos. Toxicity studies were also conducted on aquatic invertebrates. Bouly et al. (2022) investigated the influence of diclofenac on *Lymnaea stagnalis* freshwater snails with varying concentrations from the embryo stage until maturity.^80^ Diclofenac was found to have hampered the development of shell and feeding behavior during the embryo stage. The immune system and energy metabolism were also compromised as an effect of exposure to diclofenac. Zhang et al. subjected freshwater crayfish to diclofenac and observed epithelium vacuolization and tubular dilation.^81^ The diversity of natural environmental communities can be decreased from the bioaccumulation of naproxen by modifying mRNA expression and damage gastrointestinal tract and kidneys within aquatic organisms.^82^ Diclofenac can trigger the mortality of crustaceans (*Daphnia magna*) and zebrafish (*Danio rerio*).^83^

**Figure 7:**
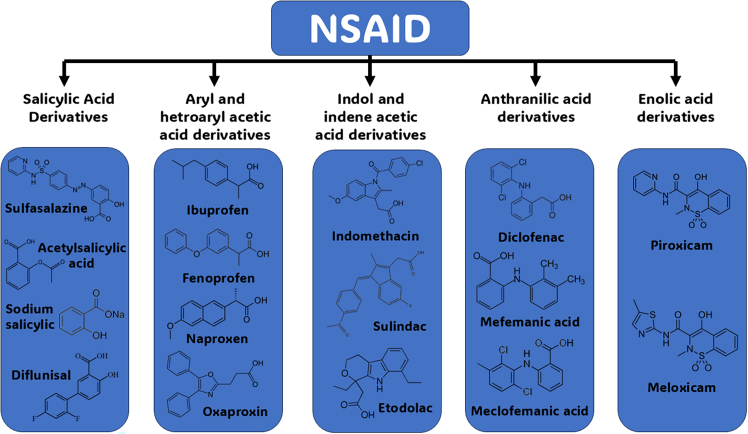
NSAIDs classification and chemical structures

**Figure 8:**
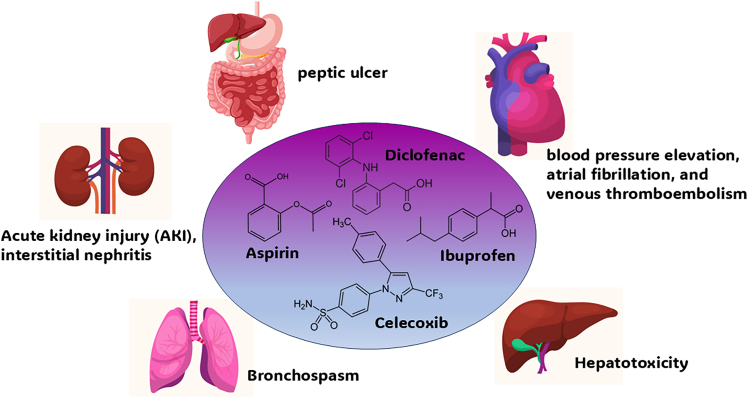
NSAIDs' impact on human body organs

NSAIDs depicted in Figure 7 are prescribed to humans as medications owing to their analgesic and anti-inflammatory properties. Regardless, potential risks lie when human organs are directly or indirectly exposed to these drugs as illustrated in Figure 8.^84^ NSAIDs can induce complications such as acute kidney injury and chronic kidney disease, which includes electrolyte imbalance, fluid retention-induced hypertension, and renal tubular acidosis.^85^^,^^86^ In ophthalmology, the topical use of NSAIDs can lead to corneal inflammation, epithelial defects, and even corneal melt.^87^ It was also found that the inhibition of COX-1 by NSAIDs can damage the gastrointestinal tract due to the reduction of prostaglandins levels in the mucosa.^88^

## Advanced oxidation processes and photocatalysis

### Advanced oxidation processes

Studies have shown that traces of the aforementioned pollutants remain in wastewater even after biological or conventional treatment methods due to them being chemically stable and resistant toward mineralization.^89^^,^^90^ Rapid industrialization has led the water contamination to be beyond the threshold of natural purification in the environment, thus calling for the development of cheap and environmentally friendly methods that can successfully ensure the removal of pollutants from contaminated water. Advanced oxidation processes (AOPs) are chemical processes that utilize hydroxyl radicals to oxidize pollutants in organic wastewater. The current challenge faced by society with increasing water pollution necessitates AOPs as tools for mineralizing recalcitrant organic pollutants that conventional methods had difficulty dealing with. AOPs primarily work with the generation of reactive oxygen species (ROS), particularly hydroxyl radicals (·OH) since they have a high redox potential of 2.8eV and are non-selective.^91^ The organic pollutants experience breakdown via different mechanisms which are dehydrogenation, combination or addition of radicals, and electron transfer.^92^ Carbon radicals are formed as radicals react with organic pollutants before further transformation into organic peroxyl radicals with oxygen molecules. The formation of other ROS ( superoxide anions and hydrogen peroxide), reacting with the radicals present in subsequent processes, leads to chemical destruction and, in some instances, mineralization.^93^ The capability of these ROS to break down a vast array of pollutants makes them highly competitive among water treatment technologies. AOPs outshine conventional methods, whereby they have a high rate of oxidation and mineralization, greatly reduced sludge production, and can be combined with conventional methods to improve overall treatment efficiency.^16^ Among examples of AOPs are the Fenton process, ozonation, and photocatalysis.

### Photocatalysis and its importance

Substantial research efforts have been dedicated to the innovation of functional nanomaterials to maximize the intake of light energy for the removal of different pollutants in wastewater, both organic and inorganic. In recent years, the concept of photocatalysis involves the process of accelerating a photoreaction with the adsorption of light (UV, visible, and IR region) by semiconductor materials (TiO_2_). The key idea of photocatalysis is the creation electron-hole pairs when the photocatalyst is irradiated, leading to the degradation or transformation of biological, organic, and inorganic compounds.

Photocatalysis can be classified as heterogeneous photocatalysis and homogeneous photocatalysis. Homogeneous photocatalysis is a process when the photocatalyst and the reactants are in similar phase. Despite improved kinetic reactions due to constant contact between molecules, the recovery of catalysts can be tedious, which impacts its sustainability and economic viability. However, its counterpart is more commonly found in environmental applications. Heterogeneous photocatalysis undergoes the phase transition of organic pollutants, followed by absorption on the surface of photo-catalytic material where redox reaction takes place, and finally, products are removed in bulk fluid from the surface.

The process after light absorption highlights the fundamentals of photocatalysis. Mechanisms of photocatalysis vary according to the wavelength of light incident on the catalyst surface (i.e., direct and indirect). Certain organic pollutants are capable of absorbing visible light, which is greater than 400nm, therefore undergoing photocatalytic degradation via a direct mechanism. Organic pollutants are first excited from the ground state to the triplet excited state. The excited dye then injects electron into the conduction band of TiO_2_, converting itself into a semi-oxidized radial cation. On the other hand, the injected electrons reduce the dissolved oxygen surrounding it into superoxide radical anion and, subsequently, hydroxyl radicals, which initiate the oxidation reaction. When a semiconductor (TiO_2_) absorbs energy greater or equivalent for the excitation of electrons, the process will proceed under indirect mechanism, whereby the photocatalyst leads the reaction due to the inability of pollutant to reach excited state under ultraviolet light (<400 nm).^22^ Photons from UV light promote an electron and the excited electron migrates from an occupied valence band of the photocatalyst to the unoccupied conduction band, a vacancy resulted forming a positively charge “hole” in the valence band. These electron hole pairs are the basis of the redox reactions as they are highly reactive. Holes initiate the oxidation of water molecules, producing hydroxyl radicals (OH^−^), which in turn mineralize the organic contaminants that are near the photocatalysts surface, while electrons in the conduction band reduce oxygen molecules, forming anionic superoxide radicals (O^2−^).^94^ Superoxide ions are also involved in the oxidation of pollutants and prevent the recombination of charge carriers. The formation of superoxide ions can also be done through the formation of hydroperoxyl radicals (HO^2−^).^95^ These hydroperoxyl radicals eventually form hydrogen peroxide (H_2_O_2_) when dissociating form highly reactive hydroxyl radicals. These ROS are pivotal in degrading a plethora of pollutants.

Unlike other advanced oxidation processes, photocatalysis can work under ambient temperatures, making it energy efficient and safe since it does not require high temperatures or pressures to operate.^96^ Photocatalytic systems can be adapted to treat wastewater of varying volumes and qualities. The process can be fine-tuned by adjusting parameters such as pH, catalyst concentration, and light intensity.^97^^,^^98^ Photocatalysis can to be considered environmentally friendly as it drastically cuts down or eliminates the need for harmful chemicals, at the same time, has the potential to achieve complete mineralization, turning organic pollutants into harmless end-products such as CO_2_ and H_2_O, setting it apart from other traditional methods that merely converts pollution from one to another.^99^

### Titanium dioxide as a photocatalyst

Important characteristics of an efficient photocatalyst includes excellent band gap, photostability, inexpensiveness, easily tunable properties, biocompatibility, non-toxicity, excellent photocatalytic activity, and many more leading to their use in water treatment process. The removal efficiency of photocatalyst depends upon type of catalyst used, water chemistry, wavelength, and the intensity of incident light as well as pH and temperature.

Amongst different semiconductor compounds, TiO_2_ as a photocatalytic material is used widely because of its inertness, low cost, versatility, highly photochemical stability, and reducing property. Anatase TiO_2_ crystal is widely used as the most efficient catalytic material than other polymorphs with high surface area for the efficient diffusion of product. The chemical property of TiO_2_ announces that it is a highly oxidizing agent that oxidizes polluting agents and can be used as a homogeneous or heterogeneous catalyst in each reaction.

However, pure TiO_2_ faces several limitations when dealing with organic wastewater. Firstly, most photocatalytic degradation occurs on the photocatalyst’s surface, and the limited availability of electron-hole pairs restricts the efficiency of TiO_2_. In addition, the tendency for electron hole pairs to recombine is high in titania, suppressing photocatalytic activity of TiO_2_.^100^ Secondly, the poor affinity of TiO_2_ photocatalyst toward hydrophobic organic pollutants in particular leads to low adsorption rate of these pollutants, thus slowing photocatalytic degradation. Thirdly, the instability of nanosized TiO_2_ causes aggregation, which hinders light from reaching active sites and consequently hampering TiO_2_ photocatalytic activity. When the concentration of TiO_2_ in a system is beyond optimal, the light will scatter extensively instead of penetrating deeper into the TiO_2_ nanoparticles, reducing TiO_2_ photoactivity.^101^ Next, it is practically challenging to recover nanosized TiO_2_ particles efficiently and safely after treating organic wastewater. TiO_2_ had been found to disperse well in a suspension and exhibits better efficiency than fixed support. Lastly, the large band gap makes TiO_2_ only suitable in the UV region.^100^ Pure TiO_2_ is deemed less energy efficient as compared to standard heterostructure photocatalysts as its decomposition process is sunlight driven as opposed to UV light. Hence, additional research is needed to enhance the physical and chemical attributes of the photocatalyst to allow for the activation of TiO_2_-based photocatalysts using visible or solar light. These limitations can be overcome through their modification and doping that decrease the band gap and enables them to absorb within the visible light region.

## Synthesis of bioderived carbon coupled with titanium dioxide

Bio-derived carbon, produced from renewable biomass sources such as plants, animals, and waste materials, has gained increasing attention for its role in environmental applications. This carbon material is highly valued for its sustainability, low cost, large surface area, and the presence of functional groups that facilitate adsorption and catalytic processes. Its utilization not only promotes waste recycling but also reduces the environmental footprint associated with traditional carbon sources.

Coupling bio-derived carbon with TiO_2_ offers a promising strategy to overcome the limitations of pure TiO_2_ in photocatalytic applications. The carbon component improves photocatalytic performance by enhancing charge separation, extending light absorption into the visible range, and increasing the active surface area for pollutant degradation. These properties significantly boost the efficiency of photocatalytic reactions.

### Synthesis techniques

#### Selection and preparation of bio-derived carbon

**Figure 9:**
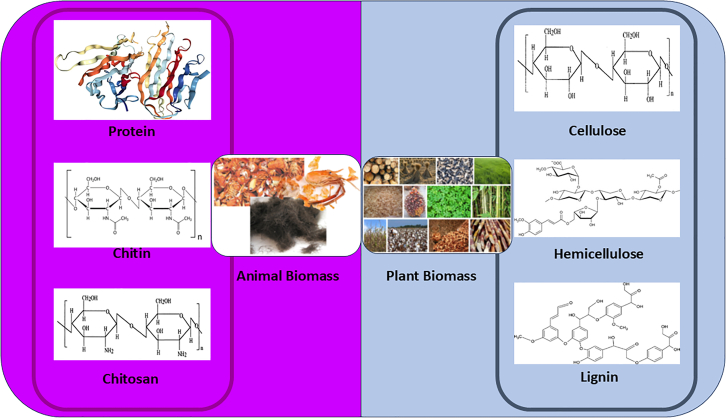
Biomass sources for carbon nanomaterials from animals and plants

The selection of biomass precursors, such as those in Figure 9 is critical for synthesizing bio-derived carbon with tailored properties for photocatalytic applications. Common sources include plant-based materials such as agricultural residues (rice husk, coconut shell, corn cob) and food waste (fruit peels, coffee grounds).^102^^,^^103^ Additionally, animal-based materials such as eggshells, fish scales, crab shells, and animal bones have also been utilized as carbon precursors due to their rich calcium carbonate or collagen content, which can be converted into carbon or carbon-based composites during thermal treatment.^104^^,^^105^^,^^106^

The preparation of bio-derived carbon generally involves thermal processes such as pyrolysis or carbonization conducted under an inert atmosphere (usually nitrogen) at temperatures between 400°C and 800°C.^107^ These processes convert biomass into carbon, retaining a porous structure that is beneficial for photocatalysis. Chemical activation using agents such as potassium hydroxide (KOH) or phosphoric acid (H_3_PO_4_) can further enhance surface area and porosity.^108^^,^^109^ For animal-based precursors, an additional deproteinization or decalcification step is often required to remove non-carbon components before pyrolysis.^109^^,^^110^

#### TiO_2_ synthesis methods

Several methods are available for synthesizing TiO_2_, each offering distinct advantages in terms of morphology, phase composition, and surface properties. Among these, the sol-gel method is widely used due to its simplicity and precise control over particle size and crystallinity.^111^ This method involves the hydrolysis and condensation of titanium precursors such as titanium isopropoxide, followed by calcination to form crystalline TiO_2_.^112^

Other methods include hydrothermal synthesis, which is effective for producing well-defined nanostructures, and chemical vapor deposition (CVD), commonly used for thin-film applications. The choice of synthesis method affects the crystalline phase (anatase, rutile, or brookite), surface area, and photocatalytic performance.

#### Coupling of bio-derived carbon with titanium dioxide

The successful integration of bio-derived carbon with TiO_2_ is crucial for enhancing photocatalytic efficiency, particularly in wastewater treatment applications. The coupling process aims to maximize the synergistic interactions between the two components, ensuring effective charge separation, increased light absorption, and improved pollutant degradation. Several methods can be employed to achieve this coupling as mentioned in Table 2, each influencing the structural, morphological, and functional properties of the final composite material.

**Table 2:** Comparison between coupling methods of bioderived carbon and TiO_2_

Coupling Method	Process Description	Advantages	Challenges
*In-Situ* Synthesis	TiO_2_ nanoparticles are grown directly on bio-derived carbon during synthesis (e.g., sol-gel, hydrothermal)	• Strong interfacial bonding • Uniform TiO_2_ distribution • Enhanced charge transfer efficiency	• Requires precise control of reaction conditions • Risk of TiO_2_ agglomeration
Post-Synthesis Mixing and Thermal Treatment	Pre-synthesized TiO_2_ and bio-derived carbon are physically mixed and subjected to heat treatment	• Simple and scalable • Independent optimization of TiO_2_ and carbon properties • Cost-effective	• Weaker interfacial bonding • Possible TiO_2_ aggregation
Hydrothermal Hybridization	TiO_2_ and bio-derived carbon are co-treated in a hydrothermal reactor under controlled temperature and pressure	• Strong chemical bonding • Enhanced material stability • Better dispersion of TiO_2_ on carbon	• Requires high-pressure equipment • Longer processing time

In *in-situ* synthesis such as sol gel and hydrothermal growth, TiO_2_ is synthesized directly on the surface of bio-derived carbon during the preparation process. The sol-gel method allows TiO_2_ precursors (such as titanium isopropoxide) to hydrolyze and condense onto the surface of bio-derived carbon, forming a homogeneous composite after calcination.^113^ For hydrothermal *in-situ* growth, bio-derived carbon is dispersed in a solution containing TiO_2_ precursors, followed by hydrothermal treatment at elevated temperatures (100°C–200°C), leading to the formation of TiO_2_ nanoparticles anchored onto the carbon matrix.^114^^,^^115^

Some researchers have opted for simpler approaches that involve post-synthesis mixing and thermal treatment, which physically mixes pre-synthesized TiO_2_ nanoparticles and bio-derived carbon, followed by heat treatment to promote adhesion and interaction between the two components.^116^^,^^117^

Lastly, some studied hydrothermal hybridization by co-treating TiO_2_ and bio-derived carbon in a hydrothermal reactor under controlled pressure and temperature.^118^^,^^119^ The process facilitates strong chemical bonding and hybridization between TiO_2_ and carbon structures.

To enhance the photocatalytic efficiency of bio-derived carbon/TiO_2_ composites, several optimization strategies must be considered. One critical factor is the carbon-to-TiO_2_ ratio, as excessive carbon content may reduce the light absorption capacity of TiO_2_, while insufficient carbon limits electron transfer.^120^^,^^121^ Typically, a balanced ratio (5–15% carbon by weight) yields the best performance.^122^ Calcination temperature is another key parameter—temperatures between 400°C and 600°C improve TiO_2_ crystallinity and interfacial bonding but may also reduce the carbon’s functional groups and porosity.^123^ Lower calcination temperatures preserve more carbon content but can compromise TiO_2_ phase purity. Surface functionalization through acid treatment (e.g., introducing -OH or -COOH groups) can enhance the interaction between TiO_2_ and carbon, improving charge separation and pollutant adsorption.^124^^,^^125^^,^^126^ Finally, optimizing reaction conditions—such as pH, precursor concentration, and reaction time—ensures a well-structured and efficient composite for environmental applications such as wastewater treatment.^127^

## Photodegradation of organic pollutants by biowaste derived carbon materials coupled with titanium dioxide

### Industrial dyes

In the past decade, the photocatalytic degradation efficiency of TiO_2_ has often been improved by coupling it with carbonaceous nanomaterials as shown in Tables 3 and 4. This enhancement is attributed to several factors, including synergistic effects, high surface area, the formation of heterostructures, and improved stability.^128^^,^^129^ In addition, the utilization of carbon nanomaterials derived from biowaste can be considered sustainable and cost effective while aiding in carbon sequestration and local waste management.^130^^,^^131^^,^^132^^,^^133^

**Table 3:** TiO_2_ coupled with biomass carbon for dye removal

Nanocomposites	Natural Resource	Synthesis Method	Dye	Experimental Conditions	Removal efficiency	Reference
TiO_2_/AC	Coffee husk	Sol gel	Victoria Blue B (VBB)	Catalyst loading = 0.5 g/L [VBB] = 15 mg/L Time = 30 min Irradiation = 18 W UV lamp	96%	Portela et al.^134^
NCQDs/TiO_2_	Spent coffee grounds	Hydrothermal-calcination	Methylene Blue (MB)	Catalyst loading = 0.1 g/L [MB] = 10 mg/L Time = 30 min Irradiation = 300 W xenon lamp	93.10%	Jin et al.^135^
CQDs/TiO_2_	Waste rice noodle (WRN)	Hydrothermal carbonization	Methylene Blue (MB)	Catalyst loading = 4 g/L [MB] = 20 mg/L Time = 80 min Irradiation = 20 W xenon lamp	99.87%	Jin et al.^136^
CQDs/TiO_2_	Waste rice noodle (WRN)	Hydrothermal carbonization	Malachite Green (MG)	Catalyst loading = 4 g/L [MG] = 20 mg/L Time = 80 min Irradiation = 20 W xenon lamp	99.00%	Jin et al.^136^
CQDs/TiO_2_	Waste rice noodle (WRN)	Hydrothermal carbonization	Methyl Violet (MV)	Catalyst loading = 4 g/L [MV] = 20 mg/L Time = 80 min Irradiation = 20 W xenon lamp	99.00%	Jin et al.^136^
CQDs/TiO_2_	Waste rice noodle (WRN)	Hydrothermal carbonization	Rhodamine (RhB)	Catalyst loading = 4 g/L [RhB] = 20 mg/L Time = 30 min Irradiation = 20 W xenon lamp	99.00%	Jin et al.^136^
TiO_2_/biochar	Nutshells	Drop casting	Methylene Blue (MB)	Catalyst loading = 1.0 g/L [MB] = 10 mg/L Time = 180 min Irradiation = UV light	90.00%	Pinna et al.^137^
TiO_2_/biochar	Microalgae (*Nannochloropsis* sp.)	Drop casting	Methylene Blue (MB)	Catalyst loading = 1.0 g/L [MB] = 10 mg/L Time = 180 min Irradiation = UV light	90.00%	Pinna et al.^137^
TiO_2_/biochar	*Salvinia molesta*	Mechanical mixing	Acid Orange 7 (AO7)	Catalyst loading = 0.1 g/L [AO7] = 20 mg/L Time = 180 min Irradiation = 15W UV light	90.00%	Silvestri et al.^138^
TiO_2_/BC	Coconut shell	Sol gel	Reactive Brilliant Blue (KN-R)	Catalyst loading = 6 g/L [KN-R] = 30 mg/L Time = 60 min pH = 1 Irradiation = 300W xenon lamp	99.71%	Zhang and Lu^139^
Bamboo charcoal/TiO_2_	Bamboo	Calcination	Methylene Blue (MB)	Catalyst loading = 0.2 g/L [MB] = 12.8 mg/L Time = 180 min Irradiation = 700W mercury lamp and 700W xenon lamp	UV light = 95% Visible light = 97%	Wang et al.^140^
AC/TiO_2_	*Manilkara Zapota*	Co-precipitation	Rh-B	Catalyst loading = 1 g/L [Rh-B] = 1 × 10^−5^ M Time = 30 min Irradiation = Solar light	91%	Parvathiraja et al.^141^
TiO_2_/GMAC	Grape marc	Impregnation	Reactive Black 5 (RB5)	Catalyst loading = 1 g/L [RB5] = 100 μmol L^−1^ Time = 70 min pH = 6.2 Irradiation = 60W UV lamp	98.93%	Belayachi et al.^142^
HS-TiO_2_ and OR TiO_2_	Hazelnut shell (HS) and Olive residue (OR)	Hydrothermal carbonization	Methylene blue (MB)	Catalyst loading = 1 g/L [MB] = 10ppm Time = 420 min pH = 6.2 Irradiation = 96W xenon lamp	96.97%	Donar et al.^143^

**Table 4:** TiO_2_ coupled with biomass carbon for pharmaceutical removal

Nanocomposites	Natural Resource	Synthesis Method	Pharmaceuticals	Experimental Conditions	Removal efficiency	Reference
Biochar/TiO_2_	Orange peel	Ultrasonication	Acetaminophen (ACT)	Catalyst loading = 0.8 g/L [ACT] = 20 mg/L Time = 100 min Irradiation = 300 W xenon lamp	94.0%	Mohtaram et al.^144^
AC/TiO_2_	Corncob	Wet precipitation	Ceftriaxone (CEF)	Catalyst loading = 1.0 g/L [CEF] = 100 mg/L Time = 240 min Irradiation = 300 W xenon lamp	99.6%	Abdullah et al.^145^
AC/TiO_2_	Date stems	*In situ* impregnation	Atenolol (AT)	Catalyst loading = 0.8 g/L [AT] = 50 mg/L Time = 120 min Irradiation = 400 W UV–mercury lamp	73%	Samir et al.^146^
AC/TiO_2_	Date stems	*In situ* impregnation	Propranolol (PR)	Catalyst loading = 0.8 g/L [PR] = 50 mg/L Time = 120 min Irradiation = 400 W UV–mercury lamp	94%	Samir et al.^146^
N-SCQDs@TiO_2_	Rice straw	Hydrothermal	Sulfadiazine (SDZ)	Catalyst loading = 0.1 g/L [SDZ] = 10 mg/L Time = 480 min Irradiation = 20 W UV lamp	99.03%	Yang et al.^147^
AC/TiO_2_	Furfural residue	Ultrasonic-assisted sol-gel and solvothermal, microwave assisted treatment	Tetracycline (TC)	Catalyst loading = 0.25 g/L [TC] = 20 mg/L Time = 120 min Irradiation = 20 W UV lamp	88%	Ao et al.^148^
AC/TiO_2_	*Argania Spinosa* tree nutshells	Temperature Impregnation	Sulfamethoxazole (SMX)	Catalyst loading = 0.1 g/L [SMX] = 50 mg/L Time = 240 min Irradiation = 300 W xenon lamp	67%	El Mouchtari et al.^149^
AC/TiO_2_	*Argania Spinosa* tree nutshells	Temperature Impregnation	Carbamazepine (CBZ)	Catalyst loading = 0.1 g/L [CBZ] = 50 mg/L Time = 240 min Irradiation = 300 W xenon lamp	85%	El Mouchtari et al.^149^
AC/TiO_2_	*Argania Spinosa* tree nutshells	Temperature Impregnation	Diclofenac (DCF)	Catalyst loading = 0.1 g/L [DCF] = 50 mg/L Time = 240 min Irradiation = 300 W xenon lamp	100%	El Mouchtari et al.^149^
TiO_2_/AC	Macadamia nut shells	Sol-gel	Tetracycline (TC)	Catalyst loading = 1.0 g/L [TC] = 50 mg/L Time = 75 min Irradiation = 18 W germicide lamp	100%	Martins et al.^150^
TiO_2_-BC	Spent coffee grounds	Pyrolysis	Diclofenac (DCF)	Catalyst loading = 1.0 g/L [DCF] = 20 mg/L Time = 120 min Irradiation = 125 W mercury lamp	90.0%	Lazarotto et al.^151^
PMBC@TiO_2_	Reed	Sol-gel	Sulfadiazine (SDZ)	Catalyst loading = 2.0 g/L [SDZ] = 5 mg/L Time = 120 min Irradiation = 25W UV lamp	94.6%	Yang et al.^152^
TiO_2_-BC	Reed straw	Sol-gel	Sulfamethoxazole (SMX)	Catalyst loading = 1.25 g/L [SMX] = 10 mg/L Time = 180 min Irradiation = 50W xenon lamp	91.3%	Zhang et al.^153^

Chen et al. had derived carbon fiber from rabbit hair waste (CRF) and studied the photocatalytic degradation of MB dye after forming a composite with TiO_2_.^154^ The hollow structure and scales on the surface of the rabbit hair provide a large surface area, making it favorable for the loading of the catalyst. The study highlights that the carbon from waste rabbit hair aids in the adsorption of dye onto the photocatalyst surface, traps and stores electrons, which prolongs the separation of photogenerated charge carriers. Nitrogen from rabbit hair narrows the bandgap of TiO_2_ and broadens the light adsorption range as nitrogen alters the connecting state of carbon atoms such as pyridinic and graphitic nitrogen to heteroatom state (pyrrolic nitrogen), increasing active sites for Ti bonding. The structure of the resulting composite possesses more micropores in addition to existing mesopores, endowing better capacity to trap light and shortens route for photogenerated charge carrier transport which suppresses their recombination.

Wu et al. on the other hand, selected grapefruit peel as a bio-template and carbon source to couple with TiO_2_ for the photodegradation of RhB, MB, and MO due to its abundance of phosphorus and potassium elements upon conversion to biochar.^155^ Biochar weakens the agglomeration of TiO_2_ and promotes organized growth on the grapefruit peel fibers. The nanopores of fibers provide active sites for rapid pollutant binding, improving adsorption and photocatalytic efficiency. The formation of graphitic carbon, proven by RAMAN and XPS, is also beneficial as it promotes the migration of photoinduced electrons, boosts separation efficiency, and leads to photocatalytic performance. However, the adsorption of sample PCT-400-550 on MO dye solution was relatively small, which should be related to the electric charge of the dye chromophore group. Under neutral conditions, the chromophores of RhB and MB were positively charged, whereas the chromophore of MO was negatively charged. Thus, it can be inferred that because the PCT-400-550 sample surface was negatively charged in the case of MO, the adsorption amount of MO was much lower than that in the case of RhB and MB. A similar study was conducted by Zhang and Lu successfully utilized biochar originating from coconut shell biochar supporting TiO_2_ for removal of Reactive Brilliant Blue (KN-R) dye via sol-gel method, which could prove to be an economical solution with positive environmental impact.^139^ HR-TEM and XRD results showed that the macropores of biochar ranging from 15 to 20μm and 2-4μm favored the anchoring of TiO_2_ without clogging the void, which will restrict the adsorption capability of biochar. Acidic and alkaline environments promote the production of hydroxyl and superoxide radicals, which result in a higher removal percentage. Anionic dyes such as KN-R dyes are attracted and adsorbed onto the surface of biochar, increasing the contact between TiO_2_ and KN-R molecules. The presence of biochar also prohibited the recombination process of h^+^ and e^−^. By employing tetrabutyl titanate and various diameters of bamboo powder, Wang et al. created visibly active bamboo biochar/TiO_2_ composite catalysts.^140^ SEM results showed that the wrinkles dispersed on the 2D surface of the charcoal fiber formed nanochannels that facilitate electrons which contribute to the suppression of electron hole combination. The bamboo biochar/TiO_2_ composites demonstrated photocatalytic activity when exposed to UV and visible light. Under UV and visible light, methylene blue (MB) was degraded by 95% and 97%, respectively, in 60 min. Another study was conducted whereby TiO_2_/graphene/bamboo carbon nanocomposites were synthesized via a facile multi-step process for the photodegradation of MB dye.^156^ The combination of graphene-like carbon and bamboo charcoal prolonged charge separation, improved adsorption of MB dye, and provided support for TiO_2_. Walnut shell was also used as low-cost support for TiO_2_ by Lu et al..^157^ The biochar composites all exhibited higher catalytic activity compared to pure TiO_2,_ with the highest removal efficiency of 83.23%. After 5 cycles, the biochar composite was able to retain its high photodegradation removal of methyl orange of 76.56%. The biochar played an effective role as an electron transporter and acceptor, thus extending the separation of electron hole pairs, which in turn enhanced the photocatalytic degradation of the composite. Ahmed S. El-Shafie et al. in ^158^ described the conversion of pistachio nutshells into biochar sorbents for the synthesis of TiO_2_-biochar composites, whereby the formation of honeycomb structured pores is beneficial for uptake of methyl orange. They also proposed that several processes drove the adsorption of dye adsorption, i) π-π stacking between aromatic rings of MO dye and biochar; ii) electrostatic interactions between MO and functional groups, and iii) hydrogen bonding.

Song et al. fabricated carbon from sawdust as green and cheap feedstock.^159^ Using sawdust precursor increased the porosity and pore size of the composite, offering more adsorption and reaction sites for organic and inorganic contaminant compounds. The enhanced light absorption of TiO_2_/C composites was mainly attributed to the sensitization of the carbon component coated on the surface of TiO_2_. As compared to P25, the binary composite is able to perform better under simulated sunlight irradiation for MB dye removal due to improved pore structure and electron transport.

Jin et al. utilize carbon quantum dots derived from waste rice noodles coupled with TiO_2_ to photodegrade different water-soluble dyes such as methylene blue, malachite green, methyl violet, basic fuchsin, and rhodamine B while being irradiated by visible light. The CQDs/TiO_2_ electrode exhibited a photocurrent over 60 times higher than the pure TiO_2_ electrode under irradiation, indicating faster charge transfer and better electron-hole pair separation. Additionally, its lower emission intensity in steady-state PL spectra compared to pure TiO_2_ suggests that CQD modification effectively reduces electron-hole recombination.^136^ In another study, the formation of Ti-*O*-C in NCQDs/TiO_2_ which carbon originated from *Pangium edule* kernel as reported by Waluyo et al. supports the claim of conducive interfacial charge transfer, thereby playing an essential role in the photodegradation performance.^160^

Bukhari et al. studied the conversion of waste scrap tires into activated carbon to study the effects of carbon on the bandgap modification of TiO_2_.^161^ The successful addition of AC positively shifted the binding energy of TiO_2_, indicating the formation of oxygen vacancies within the TiO_2_ lattice, which improved the photodegradation of Rh-B dye. To add on, Portela et al. reported that AC controlled the grain growth of TiO_2_ crystals.^134^ As summarized by Ernawati et al., AC facilitates the oxidation of organic substances on photocatalyst surfaces, with intermediates being adsorbed and further oxidized; adsorbs organic substances to accelerate the photo-oxidation process; enhances the mass transfer of pollutants to the TiO_2_ surface, boosting photocatalytic reactions; and lastly, dispersing TiO_2_ nanoparticles to prevent agglomeration during reactions and improve recyclability.^162^ Koç Keşir synthesized carbon dots from potato peels are found that when combined with titanium nanorods improved the degradation of MB dye as compared to pristine commercial TiO_2_ under UV-A and visible light irradiation, showing promise of carbon derived from natural resources in wastewater remediation.^163^

### Pharmaceutical

*Argania Spinosa* tree nutshells were calcined and then activated with phosphoric acid to produce activated carbon for AC/TiO_2_ nanocomposite for removal of pharmaceuticals such as diclofenac (DCF), carbamazepine (CBZ), and sulfamethoxazole (SMX).^149^ The highest adsorption of CBZ pollutant was observed with AC/TiO_2_ 9% since it had the least TiO_2_ in the composite and the highest surface area and pore volume among the synthesized composites. The Langmuir model is better suited for the adsorption of CBZ, which depicted the monolayer adsorption of pollutants due to new adsorption sites from Ti^4+^ as compared to pure AC. The high Q_max_ values also affirm the adsorption capability of AC/TiO_2_ composites toward pharmaceutical pollutants. Upon irradiation, AC/TiO_2_ 9% also exhibited the highest photodegradation removal for DCF at the rate of 0.715 mg/L·min followed by 0.327 and 0.185 mg/L·min for CBZ and SMX. DCF was completely removed after 4 h, making AC/TiO_2_ a promising photocatalyst for pharmaceutical pollutant removal. Similarly, activated carbon derived from macadamia nut shells was used for the decomposition of tetracycline (TC).^150^ The mesoporous structure yielded when combined with TiO_2_ allowed better charge carrier transport, which positively contributes to the photocatalytic activity of the photocatalyst. The activated carbon allowed better interaction between TC molecules and the photocatalyst and decreased the recombination of charge carriers.

Apart from that, reed was used as a raw material for biochar with modification from phosphoric acid for PMBC@TiO_2_ nanocomposite.^152^ The adsorption process was driven by electrostatic interactions, hydrogen-bond interactions, and π–π interactions. The adsorption capacity of PMBC is higher than BC due to the rougher surface, which provided a higher specific surface area while retaining the original structure. The results also revealed that the optimum ratio for PMBC to TiO_2_ is 1:3 as a decline in the degradation rate of SDZ in higher TiO_2_ mass ratio was an outcome of the deposition of excess TiO_2_ blocking the surface of PMBC. Lazarotto and his co-authors instead synthesized biochar with spent coffee beans for the photodegradation of diclofenac. The TiO_2_-BC, with a ratio of 1:1, was able to photodegrade 90% of the pollutant within 120 min, which is better than sole TiO_2_ under the same experimental conditions. The phenolic groups on the biochar surface promoted the production of reactive oxygen species, which are dominantly h^+^ and ·OH.^151^

Muangmora et al. studied the removal of caffeine with CQDs derived from coffee ground waste coupled with TiO_2_ by immobilizing them unto a fiberglass cloth in a batch reactor.^164^ CQDs acted as photosensitizers, which inject excited electrons into TiO_2_, making the photocatalyst active under visible light irradiation. The photocatalyst demonstrated removal efficiency of 80% with wastewater collected from the coffee pot cleaning process and can be regenerated by exposure to natural sunlight. Karaca et al. also employed NCQDs/TiO_2_ for the photocatalytic oxidation of tetracycline.^165^ The *Rumex crispus* L. derived CQDs accept electrons and facilitate charge carriers' transport, which in turn inhibits carrier recombination.

## Economical perspective of bio-carbon in photocatalysis

The integration of bio-carbon in photocatalysis presents significant economic advantages, particularly in sustainable and cost-effective environmental remediation technologies. Bio-carbon, derived from biomass sources such as agricultural waste, forestry residues, and organic byproducts, offers a low-cost alternative to conventional carbon-based materials such as graphene and carbon nanotubes. This cost reduction stems from the abundant availability and renewable nature of biomass feedstocks, which require minimal processing compared to synthetic carbon materials. When comparing bio-carbon with synthetic carbon materials, several economic factors come into play, including raw material cost, production expenses, scalability, and overall cost-effectiveness in photocatalytic applications. Bio-carbon is sourced from biomass waste, which is low-cost or even free in many cases, whereas synthetic carbon requires expensive precursors such as graphite or hydrocarbons, increasing production costs. Additionally, bio-carbon is produced via low-cost pyrolysis or hydrothermal carbonization, requiring less energy and simpler processing, whereas synthetic carbon involves high-temperature synthesis and chemical treatments, leading to high capital and operational expenses.

From a performance perspective, bio-carbon enhances photocatalytic efficiency by improving charge separation and surface area at a fraction of the cost, making it suitable for large-scale applications.^166^ Although synthetic carbon offers superior electronic properties, its significantly higher costs limit its feasibility for widespread use. In terms of scalability and industrial feasibility, bio-carbon is easily scalable due to the abundance of feedstocks and simple processing, aligning with circular economy principles, whereas synthetic carbon faces scalability challenges due to cost-intensive production methods.^167^ Moreover, bio-carbon provides dual benefits by utilizing waste materials and reducing environmental impact while maintaining cost-effectiveness, whereas synthetic carbon often involves environmentally hazardous processes and is less sustainable in the long term.^168^

The use of bio-carbon in photocatalysis presents an economically viable solution for environmental remediation, balancing cost-effectiveness with sustainability. By improving the efficiency of semiconductor materials such as TiO_2_, bio-carbon-modified photocatalysts can lower energy consumption, reducing operational costs in wastewater treatment plants and industrial applications.^169^ Additionally, bio-carbon supports waste valorization by converting organic waste into value-added photocatalytic materials, reducing landfill burdens, and creating economic incentives for waste management.^170^^,^^171^^,^^172^ Furthermore, bio-carbon-based photocatalysts contribute to cost reductions in advanced oxidation processes (AOPs), commonly used in water purification and air treatment. The ability to recycle and regenerate bio-carbon materials enhances their economic viability, making them a competitive alternative to synthetic carbon-based systems.^173^

From an economic standpoint, bio-carbon emerges as a more cost-effective and sustainable alternative to synthetic carbon in photocatalysis. While synthetic carbon materials may offer slightly better performance, their high production costs, and scalability challenges limit widespread adoption. In contrast, bio-carbon provides a balance of affordability, efficiency, and sustainability, making it an attractive option for large-scale environmental and industrial applications.

### Conclusion

Carbonaceous materials characteristically have large specific surface areas and porous surfaces, which make them ideal for the adsorption and photocatalytic degradation of organic pollutants. Carbonaceous materials provide active sites and oxygenated functional groups, which facilitate the binding of organic pollutants to the active sites via hydrogen bonding, n-π conjugation, ion exchange process, and so forth. In this review, different bio-originated carbon-based materials coupled with TiO_2_ composites were involved in the photocatalytic degradation of organic pollutants such as organic dyes and pharmaceuticals and their adsorption and photocatalytic degradation mechanism. The addition of carbon materials to TiO_2_ particles greatly enhanced their photodegradation efficiency. The main aim of this review was to identify potential cheaper sources for carbon precursors to treat wastewater effectively.

## Future perspective

The integration of biowaste-derived carbon materials with TiO_2_ presents a sustainable and innovative approach to environmental pollution mitigation. While significant strides have been made, numerous challenges and opportunities remain that could shape the future of this field. A critical area of focus is the scalability of synthesis methods. Developing cost-effective, low-energy, and environmentally friendly synthesis methods is essential. For example, leveraging green chemistry principles or using waste heat from industrial processes to produce biowaste-carbon composites could improve scalability without sacrificing performance.

Cutting-edge research on the industrial application of titanium dioxide coupled with biocarbon is gaining momentum. Pilot-scale studies should be prioritized to bridge the gap between laboratory success and real-world implementation. Incorporating biocarbon-TiO_2_ composites into existing industrial water treatment frameworks, such as large-scale photocatalytic reactors, would provide critical insights into their practical viability. Collaborations between academic researchers, engineers, and industries will be key to advancing these efforts.

Enhancing the visible-light activity of TiO_2_-carbon composites is another pressing challenge. Innovations such as doping with metals or non-metals or combining TiO_2_ with other semiconductors can extend the composites’ light absorption spectrum while improving charge separation efficiency. Additionally, the application of renewable light sources, such as solar energy, holds significant promise for increasing the sustainability of photocatalytic systems. Developing materials that are specifically optimized for natural sunlight conditions will further enhance the green credentials of this technology.

Future work should also explore the synergistic degradation of multiple pollutants. Real-world wastewater is often a complex mixture of contaminants, including dyes, pharmaceuticals, and other emerging pollutants. Biowaste-derived carbon-TiO_2_ composites have demonstrated the potential to tackle such mixtures, but further optimization is needed to achieve consistent and efficient degradation across a wide range of pollutants. Hybrid systems that integrate photocatalysis with adsorption or filtration technologies could enhance pollutant removal in challenging scenarios.

Recoverability and reuse of the photocatalysts remain critical areas for improvement. Developing advanced materials such as magnetic or self-separating composites would facilitate catalyst recovery, reducing secondary waste generation and improving overall sustainability.

Interdisciplinary collaboration will play a pivotal role in accelerating advancements. Combining materials science with data-driven techniques, such as machine learning, can streamline the discovery and optimization of novel composites. Moreover, partnerships with industries for pilot-scale testing and commercialization will be crucial to realizing the industrial application of these materials.

Finally, aligning future developments with global sustainability goals is imperative. The valorization of biowaste not only reduces environmental impact but also promotes circular economy principles. By integrating renewable energy sources and utilizing waste-derived materials, biocarbon-TiO_2_ composites can contribute significantly to cleaner water resources and environmental sustainability.

With ongoing innovation, collaborative efforts, and alignment with sustainability goals, the potential of biowaste-derived carbon-TiO_2_ composites to transform environmental remediation technologies is immense. Their ability to degrade complex pollutants efficiently, coupled with sustainable energy integration, positions these materials as a cornerstone for next-generation water treatment solutions.

## Acknowledgments

This work was financially supported by The Higher Institution Centre of Excellence (HiCoE) program (1000/016/018/28 Jld.3 and NANOCAT-2024D) under the 10.13039/501100002385Ministry of Higher Education, Malaysia and Universiti Malaya Research Excellence Grant 2023 (UMREG004-2023). The authors extend their appreciation to the 10.13039/501100023674Deanship of Research and Graduate Studies at King Khalid University for funding this work through Small Research Project under grant number RGP 1/210/45.

## Author contributions

Conceptualization, Ethan Dern Huang Kong and Chin Wei Lai; Writing – original draft, Ethan Dern Huang Kong and Chin Wei Lai; writing - review and editing, Ethan Dern Huang Kong and Chin Wei Lai; funding acquisition, Chin Wei Lai; resources, Chin Wei Lai; and supervision, Chin Wei Lai, Joon Ching Juan, Yean Ling Pang, Cheng Seong Khe, Irfan Anjum Badruddin, Femiana Gapsari, and Khairul Anam.

## Declaration of interests

The authors declare no competing conflicts.
